# The Regulation of TIGAR

**DOI:** 10.1186/2049-3002-2-S1-P38

**Published:** 2014-05-28

**Authors:** Pearl Lee, Eric C Cheung, Karen H Vousden

**Affiliations:** 1 The Beatson Institute for Cancer Research, Glasgow, UK

## 

The p53 tumour suppressor protein inhibits the development of cancer by initiating various cellular responses including apoptosis, senescence and cell cycle arrest. In addition to this, recent studies have found that p53 is also able to influence cell metabolism. *TP53*-induced glycolysis and apoptosis regulator (TIGAR) was discovered as a p53 target functioning as a fructose-2,6-bisphosphatase. In this way, TIGAR promotes the diversion of glycolytic metabolites to other pathways such as the pentose phosphate pathway, which allows for NADPH production to protect cells from oxidative stress as well as providing ribose-5-phosphate for nucleotide synthesis.

While discovered as a p53 target, comparison between human and mouse TIGAR suggests that mouse TIGAR is not as greatly induced by p53. In mouse intestines, TIGAR is important in repair following tissue injury and mice that are TIGAR-deficient show elevated levels of oxidative stress following damage. As the Wnt/Myc signalling pathway is important in cellular proliferation and tissue regeneration, its potential role in regulating TIGAR was investigated. Deletion of APC in mouse intestines, leading to high Wnt pathway activation, results in an increased expression of TIGAR, which is reduced when Myc is also lost, suggesting that the Wnt/Myc signalling pathway may influence the regulation of TIGAR.

Together, the results suggest that TIGAR can be regulated through p53-dependent and independent mechanisms to contribute to the control of tissue regeneration and tumorigenesis.

